# Sarcopenia in Type 2 Diabetes Mellitus: Study of the Modifiable Risk Factors Involved

**DOI:** 10.3390/jcm12175499

**Published:** 2023-08-24

**Authors:** Surapaneni Lakshmi Sravya, Jayshree Swain, Abhay Kumar Sahoo, Swayamsidha Mangaraj, Jayabhanu Kanwar, Pooja Jadhao, Srijit Das

**Affiliations:** 1 Department of Endocrinology, IMS & SUM Hospital, Bhubaneswar 751003, India; straberryeye@gmail.com (S.L.S.); drabhay76@gmail.com (A.K.S.); drsmangaraj@gmail.com (S.M.); jbk.endo@gmail.com (J.K.); poojadhao@gmail.com (P.J.); 2 Department of Human & Clinical Anatomy, College of Medicine & Health Sciences, Sultan Qaboos University, Muscat 123, Oman; drsrijit@gmail.com

**Keywords:** sarcopenia, diabetes mellitus, neuropathy, hypertension, calcium

## Abstract

(1) Background: Sarcopenia has gained much interest in recent years due to an increase in morbidity. Sarcopenia is associated with type 2 diabetes mellitus (T2DM) and vice versa. There is a paucity of information regarding the prevalence and predictors of sarcopenia among T2DM individuals. The aim of the present study was to determine the prevalence and predictors of sarcopenia among T2DM individuals. (2) Methods: This study included 159 diabetics (cases) and 79 non-diabetics (controls) aged >50 years. The subjects were assessed for demographic and anthropometric parameters. Sarcopenia (according to the Asian Working Group for Sarcopenia 2019 criteria) was assessed using Jammer’s hydraulic dynamometer for handgrip strength, dual-energy X-ray absorptiometry for muscle mass, and 6m gait speed. The biochemical investigations included glycated hemoglobin; fasting and prandial glucose; fasting insulin; lipid, renal, liver, and thyroid profiles; serum calcium; phosphorous; vitamin D; and parathyroid hormone (PTH). Appropriate statistical methods were used to determine the significance of each parameter, and a multivariate regression analysis was applied to determine the predictors. (3) Results: The prevalence of sarcopenia was significantly higher among the cases than the controls (22.5% vs. 8.86%, *p*—0.012). Body mass index (BMI) (OR—0.019, CI—0.001–0.248), physical activity (OR—0.45, CI—0.004–0.475), serum calcium levels (OR—0.155, CI—0.035–0.687), hypertension (OR—8.739, CI—1.913–39.922), and neuropathy (OR—5.57, CI—1.258–24.661) were significantly associated with sarcopenia following multivariate regression analysis. (4) Conclusions: T2DM individuals are prone to sarcopenia, especially those with a low BMI, low physical activity, hypertension, neuropathy, and low serum calcium levels. Hence, by modifying these risk factors among the elderly T2DM, sarcopenia can be prevented.

## 1. Introduction

Sarcopenia is defined as a loss of muscle mass, muscle strength, and performance [1]. Of these, strength is the most important component and is the major predictor of mortality [2]. The term sarcopenia is derived from the Greek words “sarx”, which means flesh, and “penia”, which means loss [3]. Sarcopenia is mainly a disease of the elderly due to age-related muscle loss, which is called primary sarcopenia. Secondary sarcopenia is seen with a background of systemic illness that affects muscle health [4]. Sarcopenia has gained special interest among researchers and clinicians in recent decades and has also been given a separate code in the International Classification of Disease as an independent disease (i.e., M 62.84) in the tenth revision [5]. 

The prevalence of sarcopenia varies widely across the world due to the different age groups studied, different ethnicities, associated comorbidities, and different diagnostic criteria employed. The prevalence among community-dwelling elderly individuals ranges between 9.9 to 40% [6,7], whereas among the elderly with type 2 diabetes mellitus (T2DM), it ranges between 15 to 44% [8,9,10] using various diagnostic criteria. Due to the increase in the life expectancy, approximately 2 billion elderly individuals are estimated to be diagnosed with sarcopenia by the year 2050 [11].

Sarcopenia is associated with increased morbidity and mortality due to physical disability, falls, fractures, poor quality of life, depression, and hospitalization [4]. The causes of sarcopenia are multiple, including chronic disorders like neurological disorders, inflammatory and autoimmune diseases, malnourishment, hypogonadism, any chronic systemic illness, and multiple drugs, especially glucocorticoids [12]. Amongst its various causes, diabetes is one of the most commonly encountered entity and is a common cause of sarcopenia. Impaired muscle function contributes to a sedentary lifestyle in T2DM patients, which in turn contributes to metabolic alterations, and vice versa.

Apart from age, multiple factors are involved in the causation of sarcopenia among individuals with diabetes. These include HbA1c levels, degree of insulin resistance; microvascular complications, particularly neuropathy; and macrovascular complications [13]. Other factors that are responsible for causing sarcopenia are body mass index (BMI) and physical activity [14,15].

The diagnostic criteria for sarcopenia have been evolving over the past few years. Various working groups (European, Asian, South Asian, and International) on sarcopenia have distinct cutoffs for the parameters. The three components defining sarcopenia include muscle mass, muscle strength, and functioning, while a few other criteria use only two components. A comparison of the various diagnostic criteria is summarized in Table 1.

Various studies from different parts of the world have used different criteria to define sarcopenia, and it had many cutoffs based on race and gender. Only a few Indian studies have been conducted on sarcopenia, taking subjects of variable ages with or without T2DM. Most Indian studies have used the European criteria to define sarcopenia.

These European cutoffs are higher than the Asian criteria cutoffs, thus leading to an overestimation of the prevalence of sarcopenia among Indians. In addition, the methods used to assess the components of sarcopenia were different amongst various studies. There is a paucity of studies from the Indian subcontinent on elderly T2DM investigating sarcopenia using the Asian criteria and standard methods for the assessment of the components of sarcopenia. Hence, the present cross-sectional observational study was conducted among elderly T2 DM patients using standard methods to assess sarcopenia and covering all the three aspects of sarcopenia. We assessed the prevalence using the AWGS 2019 criteria and the predictors of sarcopenia among elderly T2DM.

## 2. Materials and Methods

### 2.1. Subjects

The present cross-sectional study included individuals with T2DM aged ≥50 years as cases and age, sex, body mass index (BMI) matched healthy non-diabetic individuals as controls. The source of the samples was the outpatient Department of Endocrinology and Metabolism of our institution. Individuals with (i) secondary and type 1 diabetes, (ii) chronic kidney disease with an estimated glomerular filtration rate (eGFR) ≤ 30 mLm^2^/min (stages 4 and 5), (iii) chronic liver disease, (iv) heart failure, (v) malabsorption, (vi) malignancies, (vii) autoimmune disorders, (viii) inflammatory diseases, (ix) stroke, (x) severe hip or knee osteoarthritis (xi) cognitive impairment (xii) amputations (xiii) spinal cord injuries, (xiv) acute and chronic ongoing infections, (xv) myopathies, (xvi) BMI ≥ 40 or ≤18 kg/m^2^, (xvii) those on hormonal or nutritional supplements with high protein content, (xviii) illicit drug users, (xix) athletes, and (xx) non-consenting individuals, were excluded from the study. Both the case and control groups were subjected to clinical evaluation and detailed history taking. The sample size was calculated based on the prevalence from the previous Indian study and using the formula n = 4pq/d^2^. Permission from the ethics committee of our institution was obtained prior to the beginning of the recruitment, and informed consent was obtained from all the participants.

Each patient was asked in a detailed interview about their age, sex, duration of diabetes, past medical illness, physical activity [using the International Physical Activity Questionnaire—Short Form (IPAQ-SF) score [21]], smoking, alcohol consumption, drug history including insulin, antidiabetics, anti-hypertensives, lipid-lowering agents, and others if any. A detailed clinical examination including height, weight, BMI, and waist circumference (measured at the maximal diameter, midway between the lowest rib margin and the iliac crest during mid-expiration), were taken. Blood pressure recording and detailed systemic examinations was performed. Peripheral neuropathy was assessed through a detailed neurological examination. The Revised Neuropathy Disability Score was used to label peripheral neuropathy [22]. Pinprick sensation, vibration sensation (using a 128 Hz tuning fork), monofilament test, temperature sensation (with the cold handle of a tuning fork), and ankle reflex were assessed. A total score of six or more was labeled as diabetic peripheral neuropathy [22]. Retinopathy was assessed and classified into NPDR (non-proliferative diabetic retinopathy) and PDR (proliferative diabetic retinopathy). Nephropathy was assessed biochemically using the estimated glomerular filtration rate (eGFR) and spot urinary albumin creatinine ratio (UACR). 

Sarcopenia was evaluated using the Asian Working Group for Sarcopenia Criteria 2019 [1]. Lean body mass was measured using a dual X-ray absorptiometry (DXA) scan (GE Lunar Prodigy advance). The appendicular lean mass (ALM) was calculated as the lean body mass of all four limbs. The appendicular mass index (AMI) was calculated as ALM/Ht^2^ (in kg/m^2^). The cutoff values of <7.0 kg/m^2^ in men and <5.4 kg/m^2^ in women were taken as low muscle mass. Handgrip strength (HGS) was measured using JAMAR’s hydraulic handheld dynamometer. The dominant hand was tested. The subjects were asked to relax for 5 min and tested in a sitting position with their shoulders adducted and neutrally rotated, elbow flexed at 90°, forearm in a neutral position, and wrist slightly dorsiflexed and deviated in the ulnar direction. The subjects were asked to squeeze as hard as possible in one go and then relax. The scores of three successive trials were recorded, and an average was taken. Cutoff values of <28 kg for men and <18 kg for women were taken to describe decreased muscle strength. For gait speed, the participants were asked to stand still behind the start line and asked to walk at their normal pace until they were beyond the finish line. The walkway distance was 6 m. Timing started when the participant stepped on the start line and stopped as soon as they crossed the stop line. Their speed was calculated as distance/time in seconds. A cutoff value of <1 m/s was taken to define a decreased gait speed.

Various biochemical parameters were assessed. Fasting plasma glucose (FPG), postprandial plasma glucose (PPG), fasting lipid profile, serum calcium, serum phosphorous, serum albumin, serum alkaline phosphatase and serum creatinine, thyroid stimulating hormone (TSH) were performed by an automated analyzer. Glycated haemoglobin (HbA1c) was measured using high performance liquid chromatography as per the norms of National Glycohemoglobin Standardization Program. Sample for the serum intact parathyroid hormone (iPTH) was collected in fasting and transported in a cold chain, centrifuged and stored at −80 °C till processed and measured using electrochemiluminescence immunoassay (ECLIA). Serum 25-hydroxy vitamin D (25-OH-Vit D) and fasting insulin were measured using enzyme linked immunosorbent assay (ELISA). Insulin resistance was calculated using homeostatic model assessment for insulin resistance (HOMA-IR). 

### 2.2. Statistical Analysis

All the data analyses were carried out using the statistical product services solution IBM SPSS version 29.0. Tests of the normality assumption of continuous data were performed using an appropriate statistical test. For normally distributed continuous variables, descriptive statistics such as mean, standard deviation, and range values were calculated. Comparisons between two group means were tested using a Student’s independent t-test. For non-normal data, the median values were calculated. The median values were compared using the nonparametric Mann–Whitney U-test. Regarding categorical variables, the data were presented as frequency and percent values. Frequency data across categories were compared using a Chi-square/Fisher’s exact test as appropriate. A two-sided probability of *p* < 0.05 was considered statistically significant for all statistical tests. To study the association between various factors and sarcopenia, univariate logistic regression analysis was used first, then a multivariate logistic regression analysis was performed. 

## 3. Results

The present study included 159 individuals with T2DM as cases and 79 non-diabetic individuals as controls. The controls were age, sex, and BMImatched to the cases. The mean age of our study population was 57.4 ± 6.04 for the cases and 56 ± 5.82 for the control group. The sex distribution, male to female ratio, was 1.06 for the cases and 1.024 for the controls and mean BMI of the cases and controls were 27.4 and 26.9, respectively. The rest of the demographic parameters of the cases and controls are shown in Table 2. 

The prevalence of sarcopenia among the individuals with T2DM was 22%, which was significantly higher compared to 8.8% among the individuals without T2DM. According to the 2014 AWGS criteria, the prevalence of sarcopenia was 17.6%, which was less compared to that of 2019 criteria, which was 22%. However, this was not statistically significant. The comparison of cutoffs of the parameters of sarcopenia is shown in Table 3.

All the characteristics of the diabetic individuals, including the medications used and microvascular complications, are summarized in Table 4. The mean duration of diabetes among our diabetic group was 8.2 (±5.87) years, with 99 individuals (62.2%) having durations less than 10 years and 64 (40.2%) having 5 or fewer years of duration. Half of the study population were hypertensive, and the prevalence of dyslipidaemia was approximately 61%; however, statin intake was seen in only 49%. Approximately half (49%) of our study population had microvascular complications, of which neuropathy was the most common (36.4%), followed by nephropathy (25.7%) and retinopathy (20.7%). The mean HbA1c of our study population was 8.4%, and the mean HOMA-IR was 4.87. Approximately 82.3% had insulin resistance (HOMA-IR ≥ 2), and 61% had severe insulin resistance (HOMA-IR ≥ 3). Various antidiabetic drugs were used. A total of 73% (116) were on oral antidiabetic agents, of which metformin was the most frequently used, accounting for 90%. The majority of the cases (93.7%), were on a combination of ≥2 antidiabetic agents and insulin use in approximately 27%.

**Figure 1:**
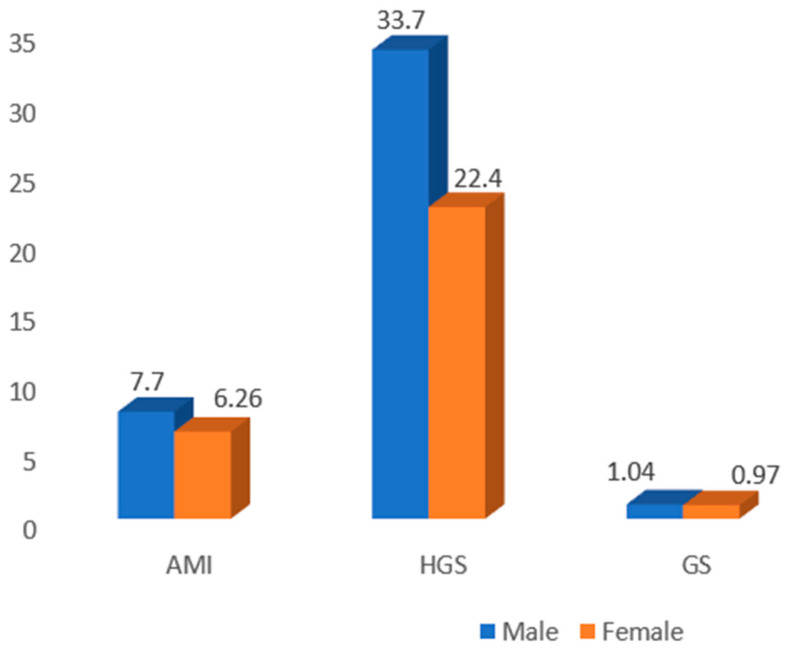
Comparison of sarcopenic parameters between the male and female diabetics.

The mean appendicular mass index (AMI), handgrip strength (HGS), and gait speed (GS) were less in females than the males, which was expected and is shown in Figure 1. The means of all the parameters were significantly low in the sarcopenic group compared to the non-sarcopenic group (Table 5). Comparisons of all the baseline parameters between the sarcopenic and non-sarcopenic diabetics are shown in Table 6.

A univariate regression analysis revealed a significant association between sarcopenia and age, alcohol intake, physical activity, BMI, waist circumference, hypertension, insulin usage, sulphonylureas, neuropathy, nephropathy, retinopathy, triglyceride levels, VLDL (very low density lipoprotein) levels, eGFR, UACR, serum calcium, and parathyroid hormone (iPTH), as shown in Table 7.

After applying the multivariate regression analysis (shown in Table 8), we found that physical activity was associated with a significant decrease in the risk of sarcopenia, with an odds ratio of 0.45 (CI of 0.004–0.475). We observed that the risk of sarcopenia was significantly lower in patients with a BMI > 27.5 kg/m^2^ (Table 8). Hypertension was significantly associated with sarcopenia, with an odds ratio of 8.739 (CI of 1.913–39.922, *p*—0.005). Of the microvascular complications, only neuropathy showed a significant positive association, with an odds ratio of 5.57 (CI of 1.258–24.66). Amongst all the biochemical parameters, only serum calcium was significantly negatively associated with sarcopenia, with an odds ratio of 0.155 (CI of 0.035–0.687), implying that the lower the serum calcium levels, the higher the risk of sarcopenia.

Other parameters like age, alcohol intake, insulin and sulphonylurea use, waist circumference, serum triglycerides, eGFR, urine ACR, serum iPTH, nephropathy, and retinopathy showed a significant association based on the univariate analysis; however, they were insignificant after the multivariate regression analysis. Lower physical activity, lower BMI, the presence of hypertension, presence of neuropathy, and low serum calcium levels were significant predictors of sarcopenia among the diabetics.

## 4. Discussion

To the best of our knowledge, this was the first study using AWGS 2019 criteria among elderly T2DM individuals in India. The prevalence of sarcopenia according to this criterion was 22% among elderly T2DM individuals compared to 8.8% among the age-, sex-, and BMI-matched healthy controls. This indicates that diabetes itself is an independent risk factor for sarcopenia. Earlier studies that followed the AWGS 2014 criteria reported a prevalence of 28.5% and 27.4%, which were slightly higher than in our study [23,24]. This difference could be due to the higher mean age of the study population. A recent Indian study reported a prevalence of sarcopenia in older diabetics (>45 years) of 31% among males and 20% among females using the AWGS 2014 criteria [25].

We also compared the 2014 and 2019 AWGS criteria cutoffs and found the prevalence of sarcopenia to be 17.6% and 22% using the 2014 and 2019 criteria, respectively. The absolute prevalence of low AMI was equal due to the same cutoffs in both criteria, whereas the absolute prevalence of low HGS and low GS was higher with the 2019 criteria, of which the prevalence of low GS was statistically significant. This was due to a change in the cutoff of HGS from 26 to 28 kgs for males and GS from 0.8m/s to 1m/s for both the sexes. 

The mean AMI in diabetic males and females was 7.75 and 6.26, respectively, which was similar (7.0 and 5.8, respectively) to an earlier report by Zengin et.al [25] among older diabetics, but less than the values reported by Kaur et al. [10] who used the bio-impedance method to assess the skeletal muscle mass index. The study population included younger diabetics as well. The mean HGS in diabetic males and females was 33.72 and 22.43, respectively, which was similar to that reported by Kaur et al [10] (32.4 and 20.6, respectively), using JAMMAR’s hydraulic hand dynamometer, where a slightly lower HGS (26.6 and 17.6, respectively) was recorded in both the sexes using a different hand dynamometer, as described by Zengin et al. [25]. The mean gait speed was 1.04 m/s and 0.97 m/s among male and female diabetics, respectively.

Demographic characteristics like age, gender, and duration of diabetes did not show a significant association with sarcopenia. These findings were similar to reports by other researchers [26]. Neither alcohol nor smoking showed a significant association with sarcopenia, which is similar to that reported by two other studies [23,26]. However, a population-based study from China showed that chronic heavy alcohol intake is associated with an increased risk of sarcopenia [27], which was not seen in our study population due to their occasional and minimal intake of alcohol. This shows that heavy alcohol intake has an effect on muscle health, but minimal amounts may not have a significant effect.

The duration of diabetes did not show any significant association with sarcopenia. This was contrary to earlier findings reported by Sazlina et al., who showed a significant association, with an odds ratio of 1.85 with a duration >10 years [23]. This difference could be due to the higher mean duration of diabetes in their study population compared to our study population (10 ± 6.6 vs. 8.2 ± 5.8 years).

In the present study, physical activity had a significant association with sarcopenia, showing that moderate physical activity decreases the risk of sarcopenia (odds ratio of 0.045), which was persistently seen after multivariate logistic regression, also with an odds ratio of 0.45. This means that with regular physical activity, the risk of sarcopenia was decreased by 45%. This finding was similar to earlier published studies [10,23]. As physical activity increases, particularly moderate or vigorous physical activity, muscle mass and strength also increase [28]. A recent Korean population-based study conducted on the elderly population (>60 years) showed that muscle mass and handgrip strength were significantly higher among men and women engaged in moderate or vigorous physical activity compared to those who had minimal physical activity, with an odds ratio of 0.77 [28]. Thus, regular physical activity is a safe strategy for preventing sarcopenia (for both loss of muscle mass and muscle strength). Physical activity also indirectly benefits muscle health by improving glycemic control [29]. Physical activity increases muscle mass and improves insulin-independent glucose uptake by the skeletal muscle, which leads to decreased glycemia and improvements in diabetes and various other complications that lead to sarcopenia [29].

In the present study, BMI and waist circumference had a significant negative association with sarcopenia. Having a BMI within the overweight or obese ranges resulted in an odds ratio of 0.154 or 0.041, respectively. Waist circumference showed an odds ratio of 0.147. This is in agreement with the study by Sazlina et al., who reported odds ratios of 0.16 and 0.03 for the overweight and obese ranges, respectively [23]. Following the multivariate analysis, only the BMI in the obese range showed a significant association with sarcopenia, with an odds ratio of 0.019, which was also in line with that reported by Sazlina et al. [23] and Rahman et al. [30], with obese BMIs having odds ratios of 0.09 and 0.081, respectively [23,30]. Two other studies analyzing the risk factors of sarcopenia also showed that those with lower BMIs had higher risks of sarcopenia [15,31]. The probable explanation for this could be that having a low BMI is an indicator of poor nourishment, which affects protein synthesis, resulting in lesser muscle mass and strength [32]. 

The presence of hypertension was significantly associated with sarcopenia, with an odds ratio of 8.7; that is, individuals with T2DM and hypertension had an 8–9 times higher risk of sarcopenia. This could be due to the synergistic effect of both diabetes and hypertension on muscle health. This finding was different from previous published studies [23,26]. Several studies showed hypertension to be an independent risk factor of sarcopenia [33]. Hypertension can damage myocytes [33]. Hypertension showed a significant positive association after a multivariate analysis [33]. Dyslipidemia and statin use showed no significant association with sarcopenia, which was similar to two earlier reports [23,26].

Among the various antidiabetic agents, we found a positive association between insulin use and sarcopenia and a negative association between sulphonylurea use and sarcopenia. On the contrary, based on multivariate logistic regression analyses, there was no significant association. None of the other antidiabetic agents, including metformin, voglibose, DPP-4 inhibitors, and SGLT-2 inhibitors, had any significant association with sarcopenia. These findings are similar to earlier findings by Sazlina et al. [23]. However, a Japanese study reported that individuals with T2DM taking insulin showed an attenuation in the progression of sarcopenia [34]. This difference could be due to the cross-sectional design of our study, which could not assess the causal relation compared to the longitudinal design of the previous study. The present study showed no significant association between the combination of antidiabetics and sarcopenia. Though not analyzed in our study, Glucagon-like peptide 1 (GLP 1) receptor analogues were shown to be detrimental to sarcopenia [35].

All three microvascular complications showed a significant positive association with sarcopenia, with odds ratios of 4.13, 3.93, and 3.68 for neuropathy, nephropathy, and retinopathy, respectively. After a multivariate analysis, only neuropathy showed a significant association with sarcopenia, with an odds of 5.57. A recent study among T2DM individuals >50 years showed that neuropathy was associated with decreased muscle strength in the lower extremities [36]. This was in contrary to an earlier report by Sazlina et al., in which they found no association between any microvascular complications and sarcopenia [23]. Neuropathy causes sarcopenia through various mechanisms, including denervation atrophy [37]. A recent meta-analysis reported a significant positive association between microangiopathies and sarcopenia, with odds of 1.7, 2.8, and 4.8, for neuropathy, nephropathy, and retinopathy, respectively [38].

Glycemic parameters such as fasting, postprandial blood glucose, and glycated hemoglobin showed no significant association with sarcopenia. These findings are similar to earlier studies that assessed HbA1c [26,39]. Sugimoto et al. reported a significant association between HbA1c and sarcopenia (particularly with low muscle mass), even after adjusting for the other co-variates [40]. This discrepancy could be due to the age and size of the study population. Sugimoto et al. [40] studied 2067 elderly diabetics, with a mean age of 68 years, contrasting with our study, which included 159 diabetics, and the mean age was 57.4. The variability in HbA1c could be small, and our study sample size, which was small compared to the others, could be inadequate to assess this association. Other studies have also reported a significant association between higher HbA1c and sarcopenia, especially with those >8.5% [41,42].

Fasting insulin and HOMA-IR (insulin resistance index) were not associated with sarcopenia. Insulin sensitivity as a predictor of sarcopenia is a less well-studied entity. The available literature shows variable results. Lee CG et al. studied non-diabetic individuals and reported that the highest quartile of insulin resistance increased the odds of losing lean mass [43]. On the other hand, Abbatecola et al. reported a negative association between insulin resistance and sarcopenia [44].

Among the serum lipids, triglycerides and VLDL showed a significant negative association based on a univariate analysis, which was not evident based on a multivariate regression analysis. Thus, in our study, none of the serum lipid levels showed a significant association with sarcopenia. This is similar to earlier research findings [23], whereas a recent study reported that VLDL had association with a low skeletal muscle mass index [45]. 

Serum albumin levels did not have a significant association with sarcopenia. This parameter has not been well-studied in earlier research studies. Sugimoto et al. reported significantly low serum albumin levels in their sarcopenic group compared to their non-sarcopenic group [40]. Renal parameters such as eGFR and albuminuria (spot urine albumin to creatinine ratio) were significantly associated with sarcopenia. eGFR showed a negative association, and the urine albumin creatinine ratio had a positive association, with an odds ratio of 0.948 and 5.91, respectively. However, after a multivariate logistic regression analysis, these parameters had no significant association, like that of nephropathy. The findings of our study are similar to previously published results by Sazlina et al. [23]. Pechmann et al. reported a significant association between albuminuria and sarcopenia, with odds ratio of 2.84; however, they found no significant association with eGFR. This difference could be due to their higher mean age of 65.6 and a higher mean duration of diabetes of 15.4 years compared to our study population (57.4 and 8.2 years, respectively). As the duration of diabetes and age increases, the risk of diabetic nephropathy increases, and thus the proportion of patients with albuminuria also increases. 

Among the bone mineral metabolic parameters, serum calcium showed a significant negative correlation, and serum intact PTH showed a significant positive correlation with sarcopenia. Serum phosphorous and 25(OH)vitamin D had no significant correlation with sarcopenia, which was similar to an earlier study that showed no association with serum vitamin D levels [30]. However, after a multivariate regression analysis, only serum calcium showed a significant negative correlation with sarcopenia. The higher the serum calcium level, the lesser is the risk, with odds ratio of 0.15. Admittedly, serum calcium as a predictor of sarcopenia did not receive significant attention earlier. However, a recent systematic review reported the role of various minerals in muscle health, and calcium intake showed a significant association with muscle health [46]. 

Calcium plays a vital role in muscle contraction, as it facilitates the effective interaction between actin and myosin muscle fibers. In striated muscle, calcium binds to troponin c on actin filaments, causing a shift in the position of troponin–tropomyosin complexes, resulting in exposure of the myosin binding sites. This allows myosin bound by ADP and inorganic phosphate (Pi) to form cross bridges with actin. The subsequent release of ADP and Pi generates the power stroke that drives muscle contraction [47]. Changes in calcium signaling can impact the regulation of contractile forces in differentiated muscle fibers. Research has shown that alterations in calcium homeostasis are associated with skeletal muscle weakness during the aging process. The sarcoplasmic reticulum has reduced calcium availability for contractions, resulting in diminished contractile force. This, coupled with an aging-related shift from faster to slower myosin/myosin light chain forms, leads to a decline in muscle power [48]. Furthermore, a study by Seo et al. demonstrated a negative correlation between calcium intake and total body fat mass, as well as a positive correlation with appendicular skeletal mass [49]. Other studies have shown a significant role of calcium intake in muscle mass [50,51]. A role of calcium in sarcopenia has been suggested via its modulation of calpains, which are cysteine proteases responsible for the regulation of key process in myogenesis. Therefore, a deficiency could potentially lead to sarcopenic outcomes [52].

In the present study, neither the duration of diabetes, the glycemic control parameters, nor the use of diabetes medications influenced the risk of sarcopenia. However, these parameters should be studied in a longitudinal design to assess their causal role in sarcopenia. 

The present study provides a few recommendations by which sarcopenia can be prevented to some extent by simple, feasible lifestyle modifications. A particular emphasis should be given by both clinicians and national policy makers on the importance of physical activity, calcium intake, adequate control of hypertension, and maintaining a healthy BMI to prevent sarcopenia.

The strengths of the present study were that it included healthy age-, sex-, and BMI-matched controls, the use of the dual X-ray absorptiometry for appendicular mass index, Jammar’s hand-held dynamometer for handgrip strength, an assessment of the effects of antidiabetic medication, and assessment of all possible biochemical parameters that are responsible for muscle health. The present study, despite being done very meticulously using standard criteria and methods to assess sarcopenia, is not completely devoid of limitations. First, this was a cross-sectional study, which does not allow for the evaluation of causal relationships. Second, the prevalence in the study could be underestimated because we excluded all the patients with other chronic systemic illnesses who are usually more prone to sarcopenia. Third, protein intake was not included because the precise amount could not be elucidated; however, the nutritional status was also reflected by the serum albumin levels, which were assessed in our study. Fourth, the results of our study may not be applicable to the general population because the study population was taken form a single tertiary care center. Hence, large population-based and long-term follow-up studies are warranted to precisely determine the risk factors. In the future, interventional trials aimed at treating sarcopenia should be conducted. 

## 5. Conclusions

The modifiable risk factors for T2DM may help for achieving better outcomes. It should not be forgotten that individuals with T2DM are more prone to sarcopenia. These include individuals with hypertension, a low BMI, less physical activity, neuropathy, and low serum calcium levels, who are at high risk and should be screened for sarcopenia. Every individual with T2DM should be educated about these modifiable risk factors and should be encouraged to maintain a healthy BMI; regular physical activity, especially muscle strengthening exercises; control of hypertension; and adequate calcium intake to prevent sarcopenia. Further longitudinal studies are warranted to assess the causal relationships between various factors and sarcopenia.

## Figures and Tables

**Table 1:** Comparison of various diagnostic criteria for sarcopenia and their cutoffs.

	Hand Grip Strength	Muscle Mass	Gait Speed
AWGS 2019 [1]	<28 for men <18 for women	ALM/Ht^2^: <7 kg/m^2^—men <5.4 kg/m^2^—women	<1 m/s
AGWS 2014 [16]	<26 for men <18 for women	ALM/Ht^2^: <7 kg/m^2^—men <5.4 kg/m^2^—women	≤0.8 m/s
EWGSOP [11]	<30 for men <20 for women	ALM/Ht^2^: <7.26 kg/m^2^—men <5.5 kg/m^2^—women	≤0.8 m/s
EWGSOP2 [17]	<27 for men <16 for women	ALM/Ht^2^: <7 kg/m^2^—men <6 kg/m^2^—women	≤0.8 m/s
FNIH [18]	<26 for men <16 for women	ALM/BMI: <0.789 kg/BMI—men <0.512 kg/BMI—women	-
IWGS [19]	-	ALM/Ht^2^: <7.23 kg/m^2^—men <5.67 kg/m^2^—women	<1 m/s
SWAG SARCO [20]	<27.5 for men <18 for women	ALM/Ht^2^: <6.11 kg/m^2^—men <4.61 kg/m^2^—women	≤0.8 m/s

**Table 2:** Comparison of parameters between the cases and controls.

Parameter	Cases (T2DM)	Controls (without T2DM)	*p*-Value
Total number (n)	159	79	-
Age (years)	57.4 (±6.04)	56 (±5.82)	0.093
Sex (male:female)	1.066	1.024	0.891
BMI (kg/m^2^)	27.4 (±3.42)	26.9 (±3.46)	0.286
Waist circumference (cms)	98.2 (±11.64)	95.8 (±10.01)	0.115
Alcohol (%)	34 (21.4%)	15 (19%)	0.703
Smoking (%)	30 (18.8%)	6 (7.6%)	0.069
Physical activity			
Minimal	86 (54.1%)	36 (45.6%)	
Moderate	73 (45.9%)	43 (54.4%)	
Vigorous	0	0	0.15
Dyslipidemia (%)	97 (61%)	51 (64.55%)	0.594
Statin use (%)	78 (49.05%)	19 (11.94%)	<0.001
Hypertension (%)	81 (50.9%)	27 (34.17%)	0.014
FPG	140.39 ± 47.78	91.23 ± 8.86	<0.001
PPG	214.92 ± 66.36	129.97 ± 14.26	<0.001
HbA1c	8.4 ± 1.90	5.29 ± 0.34	<0.001
Total cholesterol	176.62 ± 48.22	187.59 ± 40.13	0.082
Triglycerides	165.79 ± 109.68	150.70 ± 65.71	0.261
HDL	45.64 ± 13.69	47.08 ± 13.27	0.443
LDL	98.43 ± 37.97	115.09 ± 36.57	0.002
VLDL	30.94 ± 16.79	29.74 ± 12.93	0.581
Serum albumin	4.22 ± 0.51	4.24 ± 0.35	0.672
ALP	89.94 ± 30.00	86.36 ± 25.56	0.364
eGFR	83.09 ± 18.505	92.04 ± 14.502	<0.001
Serum calcium	9.55 ± 0.529	9.55 ± 0.451	0.951
Serum phosphorous	3.66 ± 0.649	3.61 ± 0.540	0.557
Serum 25-OH-vit D	24.93 ± 10.65	23.99 ± 9.48	0.506
Serum iPTH	27.28 ± 12.612	24.55 ± 9.856	0.093
TSH	2.55 ± 1.056	2.68 ± 1.054	0.399
AMI	7.03 (±1.19)	7.45 (±1.53)	0.044
HGS	28.25 (±8.42)	32.34 (±10.33)	0.002
GS	1.011 (±0.158)	1.113 (±0.16)	<0.001
Total sarcopenia	35 (22.01%)	7 (8.86%)	0.012
Severe sarcopenia	19 (11.9%)	3 (3.7%)	0.040

**Table 3:** Comparison of the 2014 and 2019 AGWS criteria cutoffs among the cases (with T2DM).

Parameter	2014 AWGS Criteria	2019 AWGS Criteria	*p*-Value
Low AMI	37 (23.2%)	37 (23.2%)	-
Low HGS	26 (16.3%)	31 (19.4%)	0.55
Low GS	21 (13.2%)	65 (40.8%)	<0.001
Sarcopenia	28 (17.6%)	35 (22%)	0.324

**Table 4:** Glycemic parameters, antidiabetics used, and microvascular complications of diabetes in the cases group.

Parameter	Values
Mean duration of diabetes	8.2 (±5.87) years
Antidiabetic agents used	
Insulin	43 (27%)
Metformin	143 (89.9%)
Sulphonylureas	81 (50.9%)
DPP4 inhibitors	104 (65.4%)
SGLT 2 inhibitors	37 (23.2%)
Alpha glucosidase inhibitors	41 (25.7%)
Thiazolidinediones	0
Combination (>1 agent)	149 (93.7%)
Statins use	78 (49.05%)
Hypertension	81 (50.9%)
Dyslipidemia	97 (61%)
Nephropathy	41 (25.7%)
Neuropathy	58 (36.4%)
Retinopathy	33 (20.7%)
Fasting insulin	13.86 (±11.25)
HOMA-IR	4.87 (±4.80)
Urine ACR	0.227 (±0.327)

**Table 5:** Comparisons of parameters of sarcopenia between the sarcopenia and no sarcopenia groups.

Muscle Parameters	Sarcopenia (*n* = 35)	No Sarcopenia (*n* = 124)	*p*-Value
AMI	5.81 (±0.80)	7.37 (±1.04)	<0.001
HS	21.84 (±6.19)	30.06 (±8.09)	<0.001
GS	0.84 (±0.12)	1.05 (±0.13)	<0.001

**Table 6:** Table showing comparisons of parameters between the sarcopenic and non-sarcopenic diabetics.

Parameter	Sarcopenia	No Sarcopenia	*p*-Value
Total number	35 (22%)	124 (78%)	
Male:female	18:17	64:60	0.985
Mean age	61 (±6.27)	56.38 (±5.59)	<0.001
Duration of diabetes	10 (±7.49)	7.69 (5.26)	0.037
Alcohol	2 (5.7%)	32 (25.8%)	0.010
Smoking	3 (8.6%)	27 (21.8%)	0.018
BMI	24.95 (±3.02)	28.09 (±3.21)	<0.001
Waist circumference	91.54 (±10.05)	100.05 (±11.40)	<0.001
Physical activity	2 (5.7%)	71 (57.3%)	<0.001
Dyslipidemia	19 (54.28%)	78 (62.9%)	0.356
Statin	20 (57.14%)	58 (46.77%)	0.279
Hypertension	26 (74.3%)	55 (44.4%)	0.002
Insulin	16 (45.7%)	27 (21.8%)	0.005
Metformin	29 (82.9%)	114 (91.9%)	0.115
Sulphonylureas	11 (31.4%)	70 (56.5%)	0.009
DPP4 inhibitors	22 (62.9%)	82 (66.1%)	0.719
SGLT-2 inhibitors	12 (34.3%)	25 (20.2%)	0.081
Alpha glucosidase inhibitors	6 (17.1%)	35 (28.2%)	0.186
Combination of antidiabetics	32 (91.4%)	117 (94.35%)	0.873
AMI	5.81 (±0.80)	7.37 (±1.04)	<0.001
HGS	21.84 (±6.19)	30.06 (±8.09)	<0.001
GS	0.84 (±0.12)	1.05 (±0.13)	<0.001
Neuropathy	22 (62.9%)	36 (29.0%)	<0.001
Nephropathy	17 (48.6%)	24 (19.4%)	<0.001
Retinopathy	14 (40.0%)	19 (15.3%)	0.001
FPG	148.68 (±53.70)	138.04 (±45.94)	0.246
PPG	215.31(±77.82)	214.81 (±63.108)	0.969
HbA1c	8.169 (±1.87)	8.467 (±1.92)	0.423
Fasting insulin	13.41 (±10.53)	13.98 (±11.48)	0.793
HOMA IR	4.90 (±4.57)	4.86 (±4.88)	0.287
Total cholesterol	166.22 (±47.98)	179.54 (±48.07)	0.150
Triglyceride	130.85 (±48.18)	175.64 (±119.86)	0.032
HDL	49.45 (±15.29)	44.56 (±13.07)	0.062
LDL	91.82 (±39.4)	100.35 (±37.503)	0.244
VLDL	25.00 (±8.82)	32.70 (±18.16)	0.017
Serum albumin	4.15 (±0.628)	4.23 (±0.48)	0.392
ALP	89.11 (±30.00)	90.17 (±30.12)	0.854
eGFR	71.51 (±14.84)	86.35 (±18.16)	<0.001
Urine ACR	0.40 (±0.43)	0.17 (±0.27)	<0.001
Serum calcium	9.35 (±0.55)	9.60 (±0.51)	0.011
Serum phosphorous	3.76 (±0.67)	3.62 (±0.64)	0.276
Serum 25-OH-vit D	24.45 (±9.48)	25.06 (±10.99)	0.766
Serum iPTH	32.24 (±17.45)	25.87 (±10.54)	0.008
TSH	2.43 (±0.86)	2.58 (±1.105)	0.454

**Table 7:** Univariate regression analysis of various demographic and anthropometric parameters with sarcopenia as the dependent variable.

Factors	Subgroup	β Estimate	Odds Ratio (Confidence Interval)	*p*-Value
Age	50–59		Ref	
	≥60	1.26	3.527 (1.62–7.67)	0.001
Sex	F		Ref	
	M	−0.007	0.993 (0.469–2.103)	0.985
Duration of diabetes	<10		Ref	
	≥10	0.541	1.71 (0.80–3.66)	0.162
Alcohol intake		−1.75	0.174 (0.039–0.768)	0.021
Smoking		−1.09	0.337 (0.09–1.18)	0.337
Physical activity	Minimal		Ref	
	Moderate	−3.096	0.045 (0.01–0.197)	<0.001
BMI	18–22.9		Ref	
	23–27.4	−1.868	0.154 (0.047–0.505)	0.002
	≥27.5	−3.186	0.0413 (0.01–0.159)	<0.001
Waist circumference	High		Ref	
	Normal	−1.92	0.147 (0.048–0.449)	<0.001
Hypertension	No		Ref	
	Yes	1.29	3.624 (1.569–8.367)	0.003
Dyslipidemia	No		Ref	
	Yes	−0.391	0.676 (0.317–1.445)	0.313
Statin use	No		Ref	
	Yes	0.417	1.517 (0.712–3.234)	0.280
Insulin	No		Ref	
	Yes	1.11	3.025 (1.373–6.666)	0.006
Metformin	No		Ref	
	Yes	−0.858	0.424 (0.142–1.26)	0.123
Sulphonylureas	No		Ref	
	Yes	−1.04	0.354 (0.159–0.785)	0.011
DPP-4 inhibitors	No		Ref	
	Yes	−0.143	0.867 (0.397–1.891)	0.719
SGLT-2 inhibitors	No		Ref	
	Yes	0.726	2.066 (0.906–4.712)	0.084
Alpha glucosidase inhibitor	No		Ref	
	Yes	−0.642	0.526 (0.201–1.377)	0.191
Combination of drugs	<3		Ref	
	≥3	−0.204	0.816 (0.373–1.784)	0.610
Neuropathy	No		Ref	
	Yes	1.42	4.137 (1.881–9.094)	<0.001
Nephropathy	No		Ref	
	Yes	1.37	3.935 (1.771–8.746)	<0.001
Retinopathy	No		Ref	
	Yes	1.30	3.684 (1.60–8.485)	0.002
FPG		0.004	1.004	
			(0.997–1.011)	0.251
PPG		1.14	1.000 (0.994–1.006)	0.969
HbA1c	<7		Ref	
	≥7	−0.238	0.788 (0.317–1.956)	0.607
Fasting insulin		−0.004	0.995	
			(0.960–1.032)	0.792
HOMA-IR	<2		Ref	
	≥2	−0.432	0.649 (0.258–1.633)	0.359
Total Cholesterol	<200		Ref	
	≥200	−0.123	0.884 (0.395–1.977)	0.764
Triglycerides	<150		Ref	
	≥150	−0.981	0.375 (0.166–0.846)	0.018
LDL	<100		Ref	
	≥100	−0.188	0.829 (0.388–1.771)	0.628
HDL		0.0233	1.023 (0.998–1.050)	0.071
VLDL		−0.0481	0.953 (0.917–0.991)	0.015
Albumin		−0.316	0.728 (0.353–1.50)	0.390
ALP		−0.0012	0.999 (0.986–1.01)	0.853
eGFR		−0.0532	0.948 (0.923–0.974)	<0.001
Urine ACR		1.78	5.918 (2.036–17.20)	0.001
TSH		−0.148	0.863 (0.588–1.27)	0.451
Serum calcium		−0.980	0.375 (0.173–0.816)	0.013
Serum phosphorous		0.327	1.386 (0.771–2.491)	0.275
Serum iPTH		0.036	1.037 (1.007–1.067)	0.013
25-OH-Vit D		−0.005	0.994 (0.958–1.032)	0.764

**Table 8:** Results of multivariate logistic regression analysis with sarcopenia as the dependent and other factors as independent variables.

Parameter	Comparison Values	β-Estimate	Odds Ratio (95% Confidence Interval)	*p*-Value
Age	<60		Ref	
	≥60	−1.138	0.320 (0.063–1.630)	0.170
Physical activity	Minimal		Ref	
	Moderate	−3.099	0.45 (0.004–0.475)	0.010
Alcohol intake	No		Ref	
	Yes	−1.773	0.170 (0.014–1.993)	0.158
Insulin use	No		Ref	
	Yes	0.183	1.201 (0.257–5.607)	0.816
Sulphonyl urea use	No		Ref	
	Yes	−1.269	0.281 (0.049–1.625)	0.156
Hypertension	No		Ref	
	Yes	1.500	8.739 (1.913–39.922)	0.005
BMI	<23		Ref	
	23–27.4	−1.865	0.155 (0.021–1.136)	0.067
	≥27.5	−3.973	0.019 (0.001–0.248)	0.003
Waist circumference	Normal		Ref	
	High	0.267	1.306 (0.164–10.425)	0.801
Triglycerides	<150		Ref	0.273
	≥150	−0.791	0.453 (0.110–1.863)	
eGFR	-	−0.034	0.966 (0.924–1.010)	0.129
PTH	-	0.008	1.008 (0.952–1.066)	0.794
Serum calcium	-	−1.865	0.155 (0.035–0.687)	0.014
Urine ACR	-	1.272	3.566 (0.089–143.657)	0.500
Neuropathy	No		Ref	
	Yes	1.717	5.570 (1.258–24.661)	0.024
Nephropathy	No		Ref	
	Yes	−0.221	1.318 (0.083–20.843)	0.845
Retinopathy	No		Ref	
	Yes	0.266	1.305 (0.141–12.034)	0.814

## Data Availability

The data presented in this study are available from the corresponding author upon request.

## Abbreviations

ADP	adenosine diphosphate
ALM	appendicular lean mass
ALP	alkaline phosphatase
AMI	appendicular mass index
AWGS	Asian Working Group for Sarcopenia
BMI	body mass index
CI	confidence interval
DPP4	Dipeptidyl peptidase
EWGSOP	European Working Group on *Sarcopenia* in Older People
FNIH	Foundation for the National Institutes of Health biomarkers consortium
FPG	fasting plasma glucose
GFR	glomerular filtration rate
GS	gait speed
HbA1C	hemoglobin A1C
HDL	high-density lipoprotein
HGS	handgrip strength
HOMA-IR	homeostatic model assessment for insulin resistance
HTN	hypertension
IWGS	International Working Group on Sarcopenia
LDL	low-density lipoprotein
OR	odds ratio
PPG	postprandial plasma glucose
PTH	parathyroid hormone
SGLT	Sodium-glucose co-transporters
SWAG SARCO	South Asian Working Action Group on SARCOpenia
T2DM	type 2 diabetes mellitus
TSH	thyroid stimulating hormone
Urine ACR	urine albumin creatinine ratio
VLDL	very low-density lipoprotein
